# Cholecystectomy promotes colon carcinogenesis by activating the Wnt signaling pathway by increasing the deoxycholic acid level

**DOI:** 10.1186/s12964-022-00890-8

**Published:** 2022-05-25

**Authors:** Yuxia Yao, Xiangji Li, Baohong Xu, Li Luo, Qingdong Guo, Xingyu Wang, Lan Sun, Zheng Zhang, Peng Li

**Affiliations:** 1 Department of Gastroenterology, Beijing Friendship Hospital, Capital Medical University, National Clinical Research Center for Digestive Disease, Beijing Digestive Disease Center, Beijing Key Laboratory for Precancerous Lesion of Digestive Disease, Beijing, 100050 People’s Republic of China; 2 Department of Gastroenterology, Beijing Luhe Hospital, Capital Medical University, Beijing, 101149 People’s Republic of China; 3 Innovation Laboratory of Terahertz Biophysics, National Innovation Institute of Defense Technology, Beijing, 100071 People’s Republic of China; 4 Department of Retroperitoneal Tumor Surgery, Peking University International Hospital, Beijing, 102206 People’s Republic of China; 5 Department of Pathology, Beijing Luhe Hospital, Capital Medical University, Beijing, 101149 People’s Republic of China

**Keywords:** Cholecystectomy, CC, Biomarkers, DCA, Wnt signaling pathway

## Abstract

**Purpose:**

Cholecystectomy (XGB) is widely recognized as a risk factor for colon cancer (CC). Continuous exposure of the colonic epithelium to deoxycholic acid (DCA) post-XGB may exert cytotoxic effects and be involved in the progression of CC. However, the functions of the XGB-induced DCA increase and the underlying mechanism remain unclear.

**Methods:**

Colitis-associated CC (CAC) mouse models constructed by AOM-DSS inducement were used to confirm the effect of XGB on the CC progression. Hematoxylin & eosin staining was performed to assess the tumor morphology of CAC mouse models tissues. Various cell biological assays including EdU, live-cell imaging, wound-healing assays, and flow cytometry for cell cycle and apoptosis were used to evaluate the effect of DCA on CC progression. The correlation among XGB, DCA, and CC and their underlying mechanisms were detected with immunohistochemistry, mass spectrometry, transcriptome sequencing, qRT-PCR, and western blotting.

**Results:**

Here we proved that XGB increased the plasma DCA level and promoted colon carcinogenesis in a colitis-associated CC mouse model. Additionally, we revealed that DCA promoted the proliferation and migration of CC cells. Further RNA sequencing showed that 120 mRNAs were upregulated, and 118 downregulated in DCA-treated CC cells versus control cells. The upregulated mRNAs were positively correlated with Wnt signaling and cell cycle-associated pathways. Moreover, DCA treatment could reduced the expression of the farnesoid X receptor (FXR) and subsequently increased the levels of β-Catenin and c-Myc in vitro and in vivo*.* Moreover, the FXR agonist GW4064 decreased the proliferation of CC cells by repressing the expression of β-catenin.

**Conclusion:**

We concluded that XGB-induced DCA exposure could promote the progression of CC by inhibiting FXR expression and enhancing the Wnt-β-catenin pathway.

**Video Abstract**

**Supplementary Information:**

The online version contains supplementary material available at 10.1186/s12964-022-00890-8.

## Introduction

With 1.9 million new cases and nearly 1 million related deaths in 2020, colon cancer (CC) ranks third in incidence and second in mortality among the main cancer types worldwide [1]. With the help of evidence-based medicine, researchers have reported many epidemiological factors contributing to the carcinogenesis of CC, such as a low folate diet, low physical activity, and abdominal fatness [2]. Noticeably, in 1977, Werner et al.[3] proposed that patients who received cholecystectomy (XGB), a very common procedure of gallbladder removal to treat cholelithiasis, and gallbladder polyps, exhibited a significantly higher risk of CC development than others. Subsequently, many clinicians and epidemiologists further confirmed the association between XGB and CC [4–6]. However, most of those reports were based on statistical analyses of correlations, and the causal relationship between XGB and CC has not been fully illustrated.

The gallbladder is the organ that stores and concentrates the bile and regulates bile acid metabolism and intestinal flora balance. XGB can alter the homeostasis of bile acid metabolism, leading to increased bile acid reabsorption and enterohepatic circulation [7, 8]. Moreover, the overload of secondary bile acids (e.g. deoxycholic acid, DCA) could induce CC carcinogenesis [9], by destroying the intestinal mucosal barrier [10, 11]. However, the direct evidence proving the mechanism by which XGB promotes CC is still lacking.

FXR was first recognized as the product of the nuclear receptor subfamily l group H member 4 (NRlH4) and exerted its function as a nuclear receptor [12, 13]. It is distributed in the organs associated with the bile acid metabolism, including the liver and intestine, which is generally activated by farnesol derivatives [14, 15]. Normal intestinal FXR activity maintains a good efflux of bile acids back into the portal vein, and controlled reuptake of bile acids into the enterocyte limits intracellular bile acid levels [16]. Activation of intestinal FXR upregulates the expression of fibroblast growth factor 19, which inhibits bile acid synthesis in hepatocytes via the activation of hepatic fibroblast growth factor receptor 4 [15]. However, several studies reported that FXR expression is significantly decreased in CC tissues, CC cell lines, and colitis-associated CC (CAC) mouse models. The expression level of FXR is reported to be negatively correlated with tumor stage, differentiation, local recurrence, and distant metastasis, and positively correlated with a good prognosis [17–19]. Bailey et al.[20] concluded that FXR silencing in colon cancer is caused by NR1H4 promoter methylation and KRAS signaling pathway inhibition. The silencing of FXR suppressed its inhibition of the Wnt-β-catenin signaling, leading to CC carcinogenesis [21]. Regretfully, no studies, to date, have clearly elucidated the relationship between XGB, DCA and FXR.

Here with a newly established XGB-CAC mouse model, we proved that XGB significantly promotes the carcinogenesis of CC. We also identified an increased DCA level in the blood of XGB mice and proved that the increased DCA could promote the proliferation and migration of CC cells by inhibiting FXR expression and enhancing the Wnt-β-catenin pathway.

## Materials and methods

### Animals and models

**Fig. 1:**
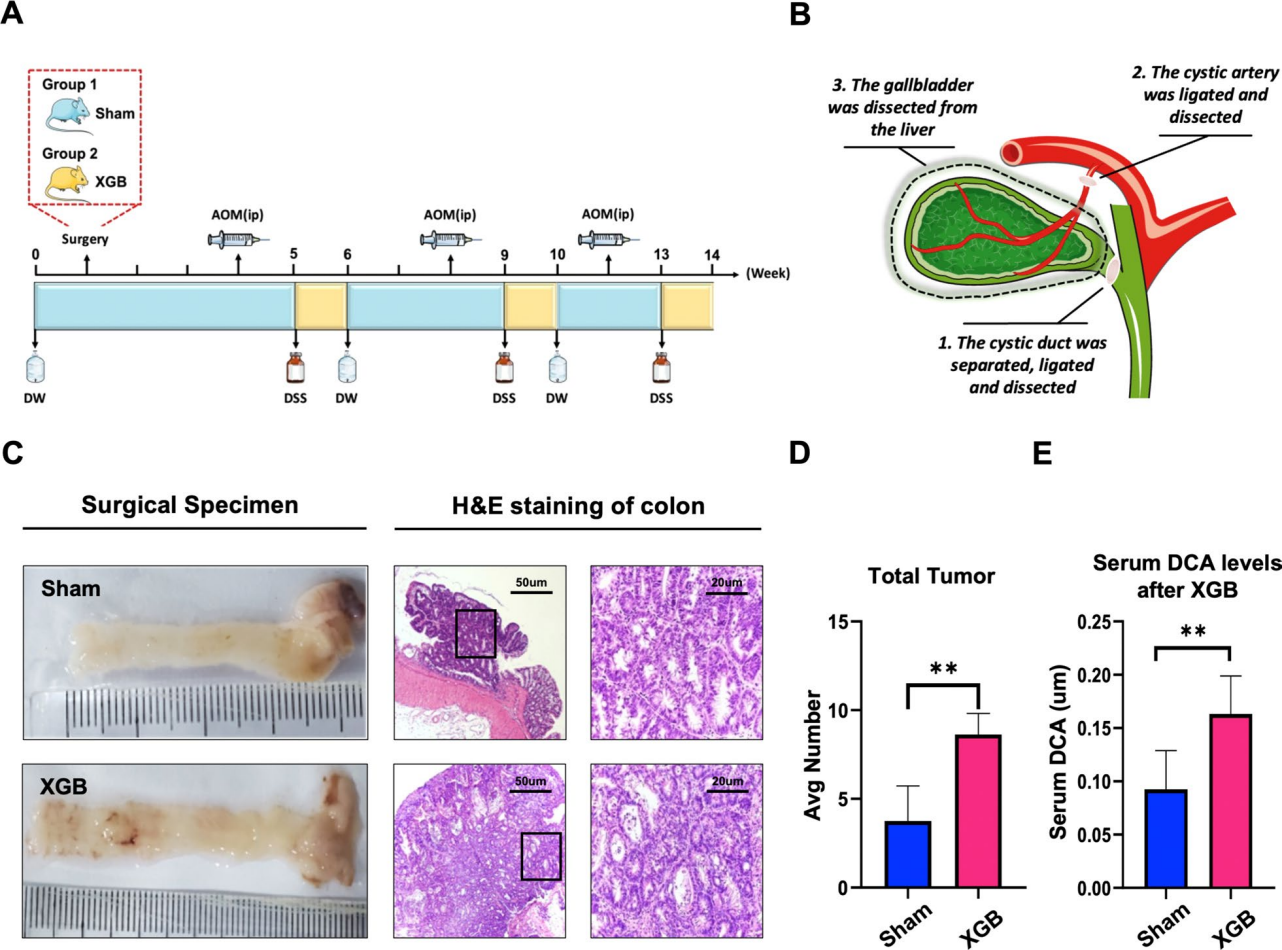
XGB-CAC mouse model construction and immunohistochemical analysis. **a** Flow chart of XGB-CAC mouse model construction. **b** Diagram of cholecystectomy. **c** H&E staining of paraffin-embedded sections of inflammatory polyp tissue (sham group) and colon cancer tissue (XGB group) was shown. The pictures right are enlargements of the rectangle in the pictures left. **d**, **e** The number of colon tumors and the level of DCA in sham and XGB group. *P < 0:05, **P < 0.01, ***P < 0.001

WT C57BL/6 male mice (n = 20) of 5 weeks of age were purchased from Beijing Vital River Laboratory Animal Technology Co. Ltd. All mice were fed specific-pathogen-free (SPF) food during the experiment. All protocols for animal studies were approved by the Institutional Animal Care Committee of Beijing Friendship Hospital Capital Medical University. After 1 week of adaptation, the mice were randomly divided into 2 groups, XGB + colon cancer and sham + colon cancer. At 6 weeks of age, all mice were injected intraperitoneally with a single dose of 10 mg/kg AOM (Sigma, A5486). After a week 2.5% dextran sulfate sodium salt (DSS) (MP Biochemicals; 36,000–50,000 Da, 0216011080) was dissolved in filtered drinking water and administered to mice for 7 days. After 2 weeks, we injected AOM and provided DSS water. This process was repeated 3 times (Fig. 1A). This dose was based on the manufacturer's instructions and literature. Colons were dissected, and polyps were counted using a stereoscope. For a subset of samples (n = 4) the distal portion of the colon was fixed in 4% paraformaldehyde, paraffin-embedded, and sectioned for hematoxylin and eosin staining. Polyps were removed from the remaining colon tissue and flash-frozen for gene sequencing. The remaining colon tissue was then divided longitudinally into two pieces for immunohistochemistry.

### XGB surgery procedures

There was no fasting before surgery so that the gallbladders were filled with bile and were found easily. Surgery was performed under isoflurane anesthesia (Isoflurane 1.5% inhalation anesthesia). The gallbladders were exposed and stripped for the sham group, and the XGB group underwent XGB after the blood vessels and cystic ducts were ligated (Fig. 1B). The incisions were sutured.

### LC–MS/MS (mass spectrometry)

Fifty microliters of orbital blood were collected for further analysis, then 200 μl of methanol homogenate containing an appropriate amount of internal standard was added, shaken at 2500 rpm for 10 min, placed in a refrigerator at − 20 °C for 10 min, and centrifuge at 12,000 rpm for 10 min. After centrifugation, the supernatant was concentrated in a concentrator and subsequently resuspend in 100ul of 50% methanol–water before a final incubation step before LC–MS/MS. The data acquisition instrument system mainly includes Ultra Performance Liquid Chromatography (UPLC) (ExionLC™ AD) and Tandem Mass Spectrometry (MS/MS) (QTRAP® 6500+).

### Histology

Paraffin block sections of the colons were stained with hematoxylin and eosin, and then evaluated blindly by two pathologists. The tumors were sequentially classified as low-grade dysplasia, high-grade dysplasia, and cancer based on the degree of proliferation.

### Immunohistochemistry

The protocol was performed as previously described. Tissue sections were incubated with the primary antibody (mouse monoclonal anti-FXR, 1:800; rabbit polyclonal anti-β-catenin,1:1500, rabbit monoclonal anti-c-Myc,1:100, rabbit monoclonal anti-cyclinD1,1:200) for 2 h at room temperature, followed by 40 min of incubation with the prediluted horseradish peroxidase-conjugated secondary antibody (1:400). For negative controls, the primary antibodies were replaced with 1% nonimmune serum in PBS. Immunohistochemistry staining was scored by measuring the integrated optical density of at least three fields of view of each slice using ImageJ 1.8 software.

### Cell culture

Human HT-29 colon cancer cells were obtained from the Cell Bank of Chinese Academy of Medical Sciences (Beijing, China) and were maintained in DMEM supplemented with 2 mM L-glutamine, 100 U/ml penicillin, 100 mg/ml streptomycin, and 10% heat-inactivated fetal bovine serum (FBS) (Gibco-BRL, Invitrogen, Paisley, United Kingdom). CaCo-2 cells (iCell Bioscience Inc.) were maintained in MEM supplemented with 1% glutamine, 1% nonessential amino acids (NEAA), 1% sodium pyruvate, and 18% FBS. Both cell types were cultured at 37 °C in a humidified atmosphere containing 5% CO_2_.

### Cell proliferation assay

Logarithmically growing CC cells were collected and counted. A total of 1,000 cells were plated in 96-well plate and cultured in 10% complete medium without DCA. For the experimental group, DCA at different concentrations (12.5, 25, 50, 100 µM) was added into 10% complete medium. A concentration of 25 µM was the best effective concentration for the three cell lines. GW4064 treatment was assessed using the same method. CC cells were treated with GW4064 (5, 10, 25, 50 μM) for 24 h according to the manufacturer’s instructions, and 10 µM was the best effective concentration. Cell proliferation was monitored for 72 h by a long-term process live-cell analysis system IncuCyte S3 (Essen Instruments, Ann Arbor, MI, United States), while the cell proliferation was assessed by confluence measurements and normalized to that at 0 h, as calculated by IncuCyte software. Photographs of the cells were taken at 12 h intervals from four separate regions per well with a 10 × objective. Values from four regions of each well were pooled and averaged across six replicates.

### Cell proliferation assay by EdU staining

Cells were incubated for 24 h in a 24-well plate after treatment. Next, the cells were incubated with 300 μl 5-ethynyl-2′-deoxyuridine (EdU) solution (50 μM) for 2 h, fixed in 4% paraformaldehyde for 30 min, and then permeabilized with 0.5% Triton X-100 for 10 min prior to staining with 300 μl Apollo staining solution for 30 min away from light. The nuclei were stained with 1 × Hoechst33342, and micrographs were taken for quantitative analyses. In this assay, The EdU staining shows red fluorescence, representing proliferating cells. The nucleus shows blue fluorescence, representing the total number of cells. We calculated the percentage of EdU-positive cells (EdU positive cell index) to represent the relative proliferation rate of all tumor cells.

### Wound-healing assays

Cells were cultured in 6-well plates until confluent (1.5% low-concentration serum was used to maintain the survival of tumor cells). Then, two artificial vertical lines were created in each well with pipette tips (10 ul) in each well. The wells were washed with phosphate-buffered saline (PBS) two times and then were cultured for an additional 48 h. The scratch lines were imaged under a microscope, and the scratch distances were measured with ImageJ 1.8 software. Each experiment was performed in triplicate。

### Cell cycle and apoptosis determination

HT-29 and CaCo-2 cells were processed and stimulated with DCA for 24 h; single-cell suspensions were prepared as previously described. For intracellular cytokine staining, cells were fixed and permeabilized by using a fixation and permeabilization solution respectively (00-5523, Bioscience). Flow cytometry data were acquired on an LSRII flow cytometer (BD Biosciences) and analyzed with ModFit LT 3.2 software.

After the corresponding treatment, the cells were subjected to fluorescein isothiocyanate (FITC)-conjugated Annexin V and propidium iodide (PI) staining, and apoptotic cells were analyzed by FACS as previously described [22].

### Transcriptome sequencing

Sample collection and preparation RNA quantification and qualification RNA integrity were assessed using the RNA Nano 6000 Assay Kit of the Bioanalyzer 2100 system (Agilent Technologies, CA, USA). Fragmentation was carried out using divalent cations under elevated temperature in First Strand Synthesis Reaction Buffer (5×). To preferentially select cDNA fragments of preferentially 370 ~ 420 bp in length, the library fragments were purified with an AMPure XP system (Beckman Coulter, Beverly, USA). PCR was performed with Phusion High-Fidelity DNA polymerase, Universal PCR primers and Index (X) Primer. PCR products were purified and library quality was assessed on the Agilent Bioanalyzer 2100 system. The clustering of the index-coded samples was performed on a cBot Cluster Generation System using TruSeq PE Cluster Kit v3-cBot-HS (Illumia) according to the manufacturer’s instructions. After cluster generation, the library preparations were sequenced on an Illumina Novaseq platform and 150 bp paired-end reads were generated [23].

### Quantitative real-time PCR (qRT-PCR) analysis

qRT-PCR assays were carried out as previously described. The relative expression of FXR was normalized to that of GAPDH using the comparative CT method as the manufacturer’s instructions (ABI, USA). All primers were designed online using NCBI Primer, and were synthesized by Shanghai Sangon Bioengineering Co. Ltd. The primers used in PCR assays were as follows (Table 1).

**Table 1:** Sequences of primers used in this study

Primer	Sequence
GAPDH (human)	Forward 5'- GGAGCGAGATCCCTCCAAAAT-3'
	Reverse 5'- GGCTGTTGTCATACTTCTCATGG-3'
FXR (human)	Forward 5'- GACTTTGGACCATGAAGACCAG -3'
	Reverse 5'- GCCCAGACGGAAGTTTCTTATT-3'
β-catenin(human)	Forward 5'-AAAGCGGCTGTTAGTCACTGG-3'
	Reverse 5'-CGAGTCATTGCATACTGTCCAT-3'
C-Myc(human)	Forward 5'-GAAGATGGTGATGGGATTT-3'
	Reverse 5'-CCACAGCAAACCTCCTCACA-3'
cyclinD1(human)	Forward 5'-GCTGCGAAGTGGAAACCATC-3'
	Reverse 5'-CCTCCTTCTGCACACATTTGAA-3'
Axin2 (human)	Forward 5'-GGAGCGAGATCCCTCCAAAAT-3'
	Reverse 5'-GGCTGTTGTCATACTTCTCATGG-3'
GAPDH (mouse)	Forward 5'- AGCTTCGGCACATATTTCATCTG-3'
	Reverse 5'- CGTTCACTCCCATGACAAACA-3'
FXR (mouse)	Forward 5'- GGCAGAATCTGGATTTGGAATCG-3'
	Reverse 5-' GCCCAGGTTGGAATAGTAAGACG-3'
β-catenin(mouse)	Forward 5-' ATGGAGCCGGACAGAAAAGC-3'
	Reverse 5-' TGGGAGGTGTCAACATCTTCTT-3'
C-Myc(mouse)	Forward 5-'ATGCCCCTCAACGTGAACTTC-3'
	Reverse 5-'GTCGCAGATGAAATAGGGCTG-3'
cyclinD1(mouse)	Forward 5-'GCGTACCCTGACACCAATCTC-3'
	Reverse 5-'ACTTGAAGTAAGATACGGAGGGC-3'
Axin2 (mouse)	Forward 5-'ATGAGTAGCGCCGTGTTAGTG-3'
	Reverse. 5-'GGGCATAGGTTTGGTGGACT-3'

All the qRT-PCR experiments were repeated at least three times with statistical analyses for each individual experimental set. Data analyses for the gene expression were performed using the 2^−ΔΔCt^ method. All values in the experiments are expressed as mean ± SEM. For the detailed protocol and data processing methods, refer to our previous publication [23].

### Western blotting

The cells were extracted using RIPA lysis buffer (Tris–HCL 50 mM, NaCl 150 mM, 1% NP-40, 0.5% sodium deoxycholate, 0.1% SDS) and disrupted with gentle sonication (on 3 s/off 3 s, 10 cycles). The protein concentration was confirmed by the BCA Protein Assay Kit (Thermo Fisher) and equilibrated with 5 loading buffer and ddH2O. The total denatured protein was separated by 10% SDS-PAGE and then transferred to a nitrocellulose membrane. Nonspecific binding sites were blocked with 5% (w/v) milk (skimmed milk powder in TBST) at room temperature for 2 h. The protein was incubated with the primary antibody overnight at 4℃. After washing 6 times with TBST, the blot was incubated with the second HRP-conjugated antibody for 2 h at room temperature and blotted with an enhanced chemiluminescence system (BIO-RAD, USA). The antibodies used were anti FXR (D3) (Santa Cruz Biotechnology, SC-25309 R), anti-β-catenin (Proteintech, 51067-2-AP), anti-c-Myc (Abcam, ab32072), anti-cyclinD1(Cell Signaling Technology, 92G2), anti-VEGF-A(Cell Signaling Technology, #65373), anti-CDK1 (Proteintech, 19532-1-AP) and anti-GAPDH (Proteintech, 60004-1-Ig). Bands of interest were distinguished according to the protein ladder molecular weight. These membranes were exposed to a chemiluminescence solution (SuperSignal West Pico Chemiluminescent Substrate, Pierce), and band intensities were revealed by optical densitometry of developed autoradiographs or by a ChemiDoc MP system (BioRad).

### Statistical analysis

GraphPad Prism 8.0 software was used to perform the statistical analyses. Data for at least three independent experiments performed in triplicate are expressed as the mean ± standard deviation (SD). We performed the homogeneity test of variance with GraphPad Prism 8.0 and all data passed the homogeneity test (*P* > 0.10). The one-sample Kolmogorov–Smirnov test was used to test whether the data was normally distributed. Unpaired, two-sided Student’s t test was used for statistical comparisons between different groups for normally distributed quantitative data. The nonparametric Mann–Whitney test was performed for the comparisons between the data with a skewed distribution data. *P* values < 0.05 were considered to indicate statistically significance.

## Results

### XGB increases plasma DCA and the progression of colon tumors in a CAC mouse model

To evaluate the effects of XGB on tumor progression, we constructed a CAC model by AOM-DSS induction in C57B/L6 mice that underwent XGB or sham surgery 3 weeks prior (Fig. 1a, b). Tumor load (including tumor number and tumor size) was significantly increased in the XGB group as compared to the sham surgery group (Fig. 1c, d). In addition, we also found that XGB led to epithelial hyperproliferation, hyperplastic crypts, and crypt-like invaginations in the villi throughout the colon (Fig. 1c). Moreover, the plasma level of DCA was significantly increased in the XGB group as compared to the sham surgery group (Fig. 1e). Together, these results proved that XGB promotes the progression of CC, which could be possibly associated with abnormal DCA metabolism.

### DCA promotes cell proliferation, cell cycle progression, and cell migration

**Fig. 2:**
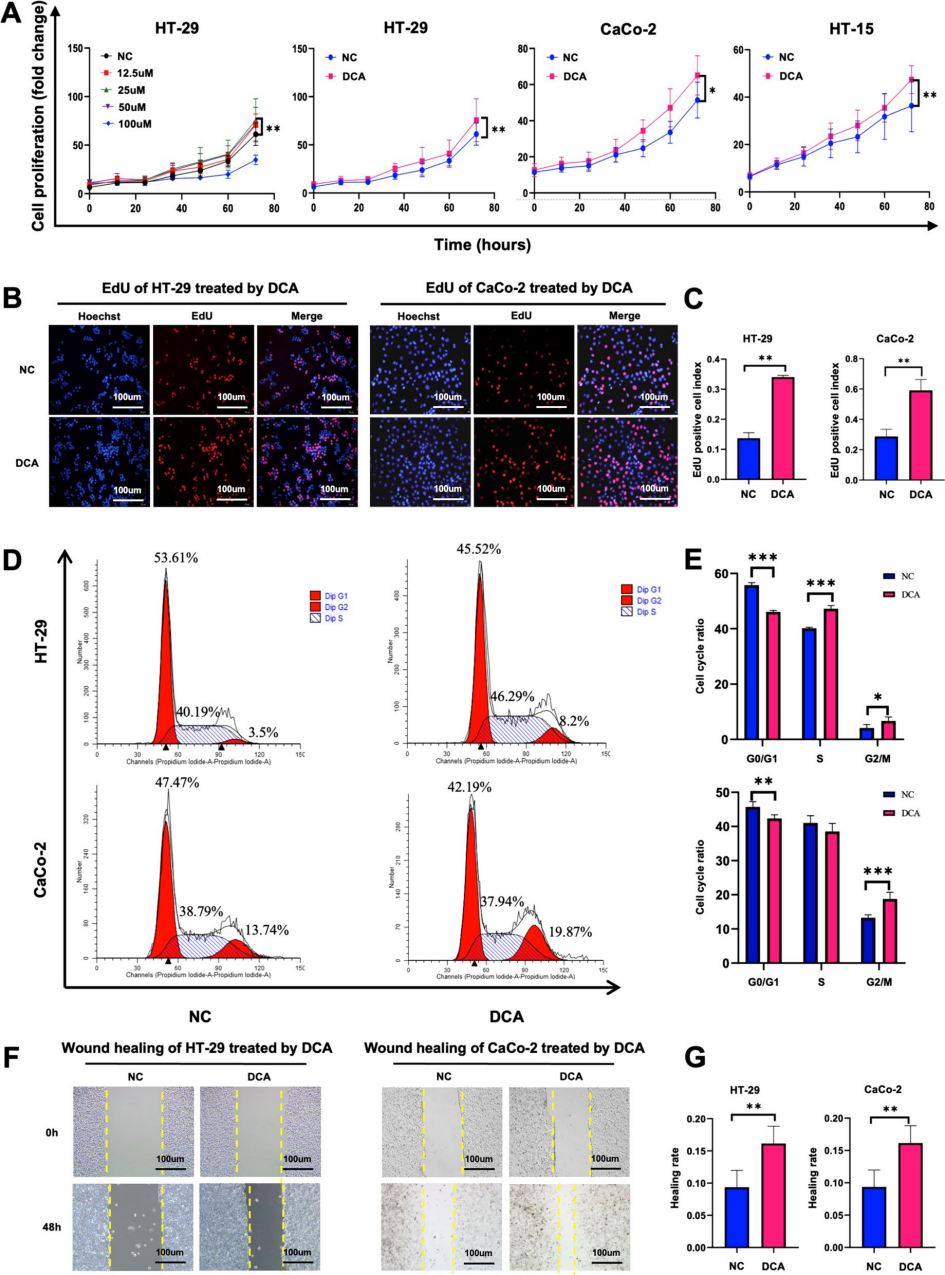
DCA promotes the proliferation, G1/S phase transition, and migration of colon cancer cells. **a**, **b** DCA promoted the viability of the CC cells in a dose- and time-dependent manner. Living-cell image and EdU were used to detect the proliferate capability of CC cells. **c** The quantitative analysis of the EdU assay. **d**, **e** FACS cell cycle assay was performed to detect the proportion of the S phase in CC cells. **f**, **g** A wound-healing assay was performed and detected at 0 h and 48 h. *P < 0:05, **P < 0.01, ***P < 0.001

To determine the effects of DCA on CC cell proliferation, different concentrations of DCA (12.5, 25, 50, 100 µM) were added to the culture medium of HT-29 cells. Considering that 25 µM DCA showed the best-promoting effects on cell proliferation, we performed subsequent analyses with 25 µM DCA in different CC cell lines. The results demonstrated that DCA increased cell proliferation by 14.34% in HT-29 cells (*P* < 0.05), by 13.7% CaCo-2 cells (*P* < 0.05) and by 10.98% HCT-15 cells (*P* = 0.04, Fig. 2a).

To assess whether the proliferation induced by DCA is associated with cell cycle progression, EdU and FACS cell cycle assays were performed. EdU assays showed that DCA significantly increased the EdU synthesis in S-phase by 20.31% in HT-29 cells (*P* = 0.001), and by 30.32% in CaCo-2 cells (*P* = 0.0139) (Fig. 2b, c). For the FACS assay, DCA decreased the proportion of Diploid G1 phase cells by 9.69% (*P* < 0.001) and increased the proportion of Diploid S phase cells by 7.10% (*P* < 0.001) and that of Diploid G2 phase cells by 2.59% (*P* = 0.013) for the HT-29 cells. The G1/S and S/G2 phases showed similar increases facilitated by DCA were also observed in CaCo-2 cells (Fig. 2d, e). In addition, we also examined the rescue effect of GW4064, a FXR agonist, on DCA treatment in CC line cells. The results indicated that GW4064 significantly inhibited the proliferation of CC line cells treated DCA (Fig. 6a, b). These results indicated that DCA could promote cell proliferation and affect the cell cycle.

We also performed cell scratch assays to investigate the effects of DCA on tumor migration. Compared to that in the control group, the healing area in the DCA group increased by 6.80% in HT-29 cells (*P* = 0.002) and by 41.80% in CaCo-2 cells (*P* = 0.001) (Fig. 2f, g) with DCA treatment for 48 h.

### DCA activates the Wnt signaling pathway and suppresses the G2/M checkpoint

**Fig. 3:**
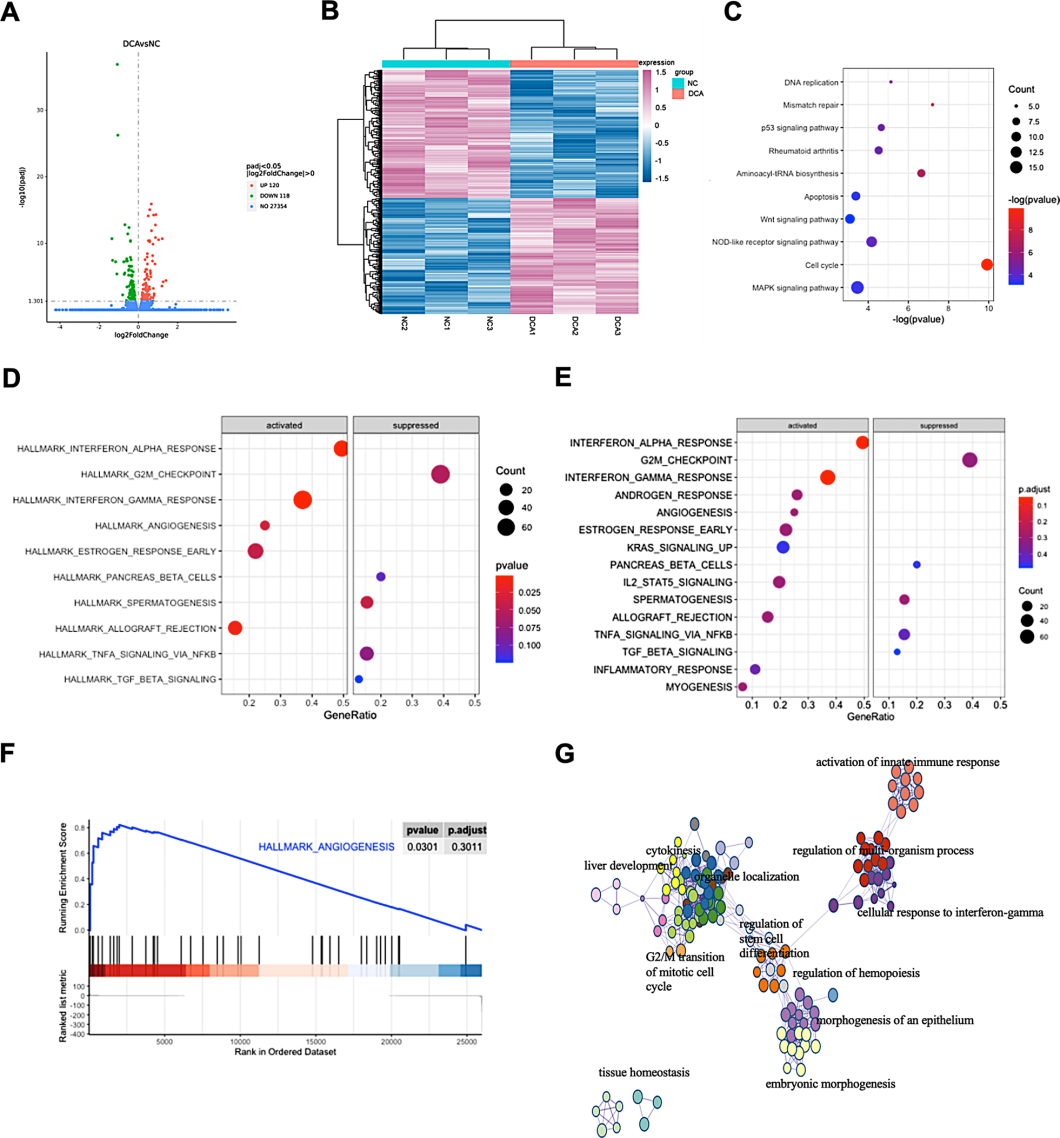
DCA activates Wnt signaling pathway and suppressed the G2/M checkpoint. **a**, **b** the DEGs of DCA vs control group were displayed by volcano and heat maps. **c**–**e** The KEGG, GESA, and Reactome analyses for DEGs. **f** Gene set enrichment analysis indicated angiogenesis-associated genes was upregulated in DCA group. **g** The PPI network among DEGs was visualized by Cytoscape and divided all DEGs into 12 MCODE functional modules

To investigate the molecular basis of the tumor-promoting effects of DCA, we performed RNA sequencing of HT-29 cells treated with 25 µM DCA. There were 120 upregulated genes and 118 downregulated in the DCA group compared with the sham group (Fig. 3a, b). Then, functional enrichment analysis was performed on those differentially expressed genes (DEGs). Kyoto Encyclopedia of Genes and Genomes (KEGG) enrichment analysis indicated that the functions of those DEGs were associated with the Wnt signaling pathway and the cell cycle (Fig. 3c). Gene Set Enrichment Analysis (GSEA) analysis showed that angiogenesis genes were activated whereas G2/M checkpoint genes were suppressed in the DCA group (Fig. 3d, f). The protein–protein interaction (PPI) network among those DEGs was visualized by Cytoscape, and all DEGs were divided into 12 MCODE functional modules (Fig. 3g).

### The XGB-induced increase in DCA levels activates the Wnt signaling pathway and suppresses the expression of FXR

**Fig. 4:**
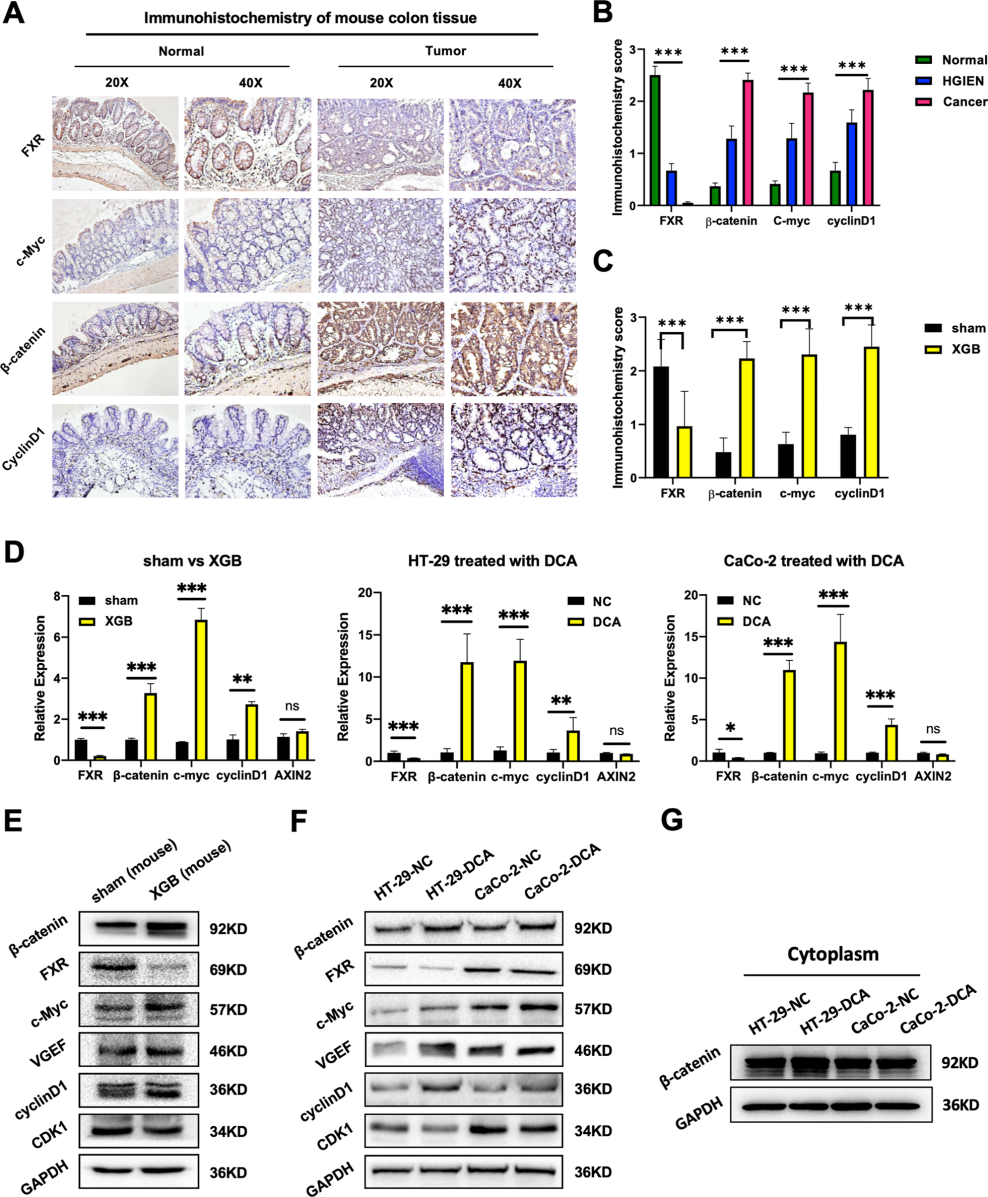
XGB-induced DCA increased activates Wnt-β-catenin signal pathway and suppressed the expression of FXR. **a**–**c** IHC staining of paraffin-embedded sections of normal and colon tissues was shown. The right pictures are the quantitative analysis of FXR, β-catenin, c-Myc, and cyclinD1 proteins in tissues (Normal, HGIEN, and Colon cancer) and groups (sham and XGB). **d** qRT-PCR was used to detect the mRNA level of FXR, β-catenin, c-Myc, cyclinD1, and Axin2 in sham and XGB groups and different CC cells, and the expression level of FXR, β-catenin, c-Myc, and cyclinD1 were higher in XGB group and DCA-treated CC cells than sham group and control cells. **e**, **f** Western blotting was used to detect the protein level of β-catenin, FXR, c-Myc, VGEF, cyclinD1, and CDK1 in sham and XGB groups and different CC cells. **g** Western blotting was used to detect the protein level of cytosolic β-catenin in different CC cells. *P < 0:05, **P < 0.01, ***P < 0.001

To verify the cell line-based RNA sequencing results in mouse models, we performed IHC analysis to evaluate the effects of XGB on the Wnt signaling pathway in cancerous (XGB group) and noncancerous colon tissues (sham group). The results showed that β-catenin and its downstream cyclin D1 were both significantly upregulated in CC tissues compared to noncancerous colon tissues (*P* < 0.001, Fig. 4a, b). Moreover, the overall IHC scores of β-catenin and cyclin D1 in the XGB group were significantly higher than those in the sham group (*P* < 0.001, Fig. 4c).

Considering FXR is a bile acid homeostasis-associated protein that can regulate Wnt signaling, we then compared the expression of FXR in the colon tissues of the XGB group and the sham group. IHC analysis demonstrated that the level of FXR was obviously reduced in CC tissues compared to noncancerous colon tissues (*P* < 0.001, Fig. 4b). Moreover, the IHC scores of FXR in the XGB group were significantly lower than those in the sham group (*P* < 0.001, Fig. 4c).

Then the expression of β-catenin, VEGF-A, the G2/M checkpoint key enzymes CDK1, and FXR in CC lines upon DCA treatment was detected by RT-PCR and Western blotting. The results were consistent with the above in vivo assays. FXR and CDK1 levels were significantly decreased, while the β-catenin level was increased in CC cell lines treated with DCA (Fig. 4d, f). To further validate Wnt-β-catenin signaling activation, we also performed cytoplasmic and nuclear fractionation, then WB was used to detect the level of β-catenin. The results showed that cytosolic β-catenin was significantly increased in HT-29 treated with DCA compared to the control group. However, it was no obvious alteration between CaCo-2-DCA and the control group (Fig. 4g). Moreover, GW4064 rescue experiment showed that cytosolic β-catenin was decreased in HT29 treated with DCA and GW4064 compared to HT-29 treated with DCA (Fig. 6e). Taking the above results, here we inferred that DCA promotes cell proliferation by repressing FXR expression and activating the Wnt-β-catenin pathway. In addition, given that Axin2 was a well-established Wnt/β-catenin target gene in the gastrointestinal tract, we performed qPCR in CC lines treated with DCA and tissues of CAC mouse model. However, the relative expression of Axin2 was only slighted elevated by XGB group (Fig. 4d). Therefore, we suggest that the target gene of β-catenin in this study model is not Axin2.

### An FXR agonist promotes cell apoptosis by downregulating the Wnt signaling pathway

**Fig. 5:**
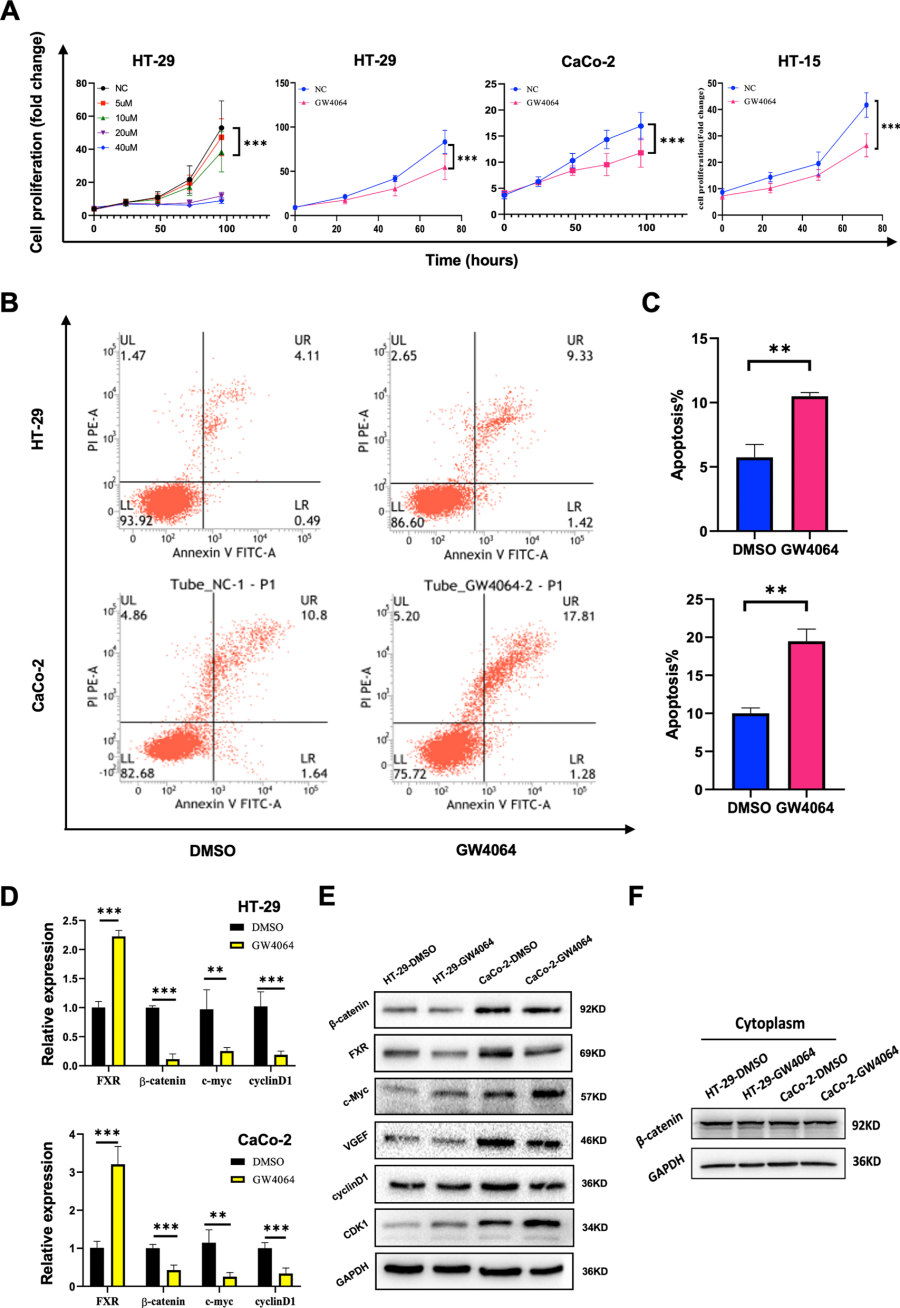
GW4064 inhibited the proliferation and apoptosis of CC cells via suppressing the Wnt-β-catenin signal pathway and FXR expression. **a** GW4064 inhibited the viability of the CC cells in a dose- and time-dependent manner. Living-cell image was used to detect the proliferative capability of CC cells. **b**, **c** Flow cytometry was performed to assess the apoptosis rate of different CC cells, and the apoptosis rate was higher in GW4064 than DMSO group. **d** qRT-PCR was used to detect the mRNA level of FXR, β-catenin, c-Myc, and cyclinD1 in different CC cells, and the expression level of FXR, β-catenin, c-Myc, and cyclinD1 were higher in DMSO than GW4064 groups. **e** Western blotting was used to detect the protein level of β-catenin, FXR, c-Myc, VGEF, cyclinD1, and CDK1 in different CC cells. **f** Western blotting was used to detect the protein level of cytosolic β-catenin in different CC cells. *P < 0:05, **P < 0.01, ***P < 0.001

We evaluated the proliferation and apoptosis rates of different CC cell lines after treatment with the FXR agonist GW4064 treatment. The effects of GW4064 on CC cell proliferation and apoptosis were evaluated. The results indicated that GW4064 inhibited cell proliferation (Fig. 5a) and promoted cell apoptosis as compared to that in the solvent (DMSO) control group (10.493 ± 0.52 versus 5.747 ± 0.44, *P* = 0.0087, 19.467 ± 1.84.52 versus 10.003 ± 1.37, *P* = 0.0088; Fig. 5b, c). To explore whether FXR promotes cell apoptosis through downregulation of the Wnt signaling pathway, the expression level of the Wnt signaling pathway components was further determined by WB and RT-PCR. The results showed that FXR was actually activated by its agonist GW4064. Moreover, the expression of Wnt-β-catenin signaling pathway biomarkers and VEGF-A was obviously decreased after GW4064 treatment (Fig. 5d, e). Besides, cytosolic β-catenin was also detected, and the results indicated that a decreasing trend of cytosolic β-catenin was observed in GW4064 groups compared to DMSO groups (Fig. 5f).

**Fig. 6:**
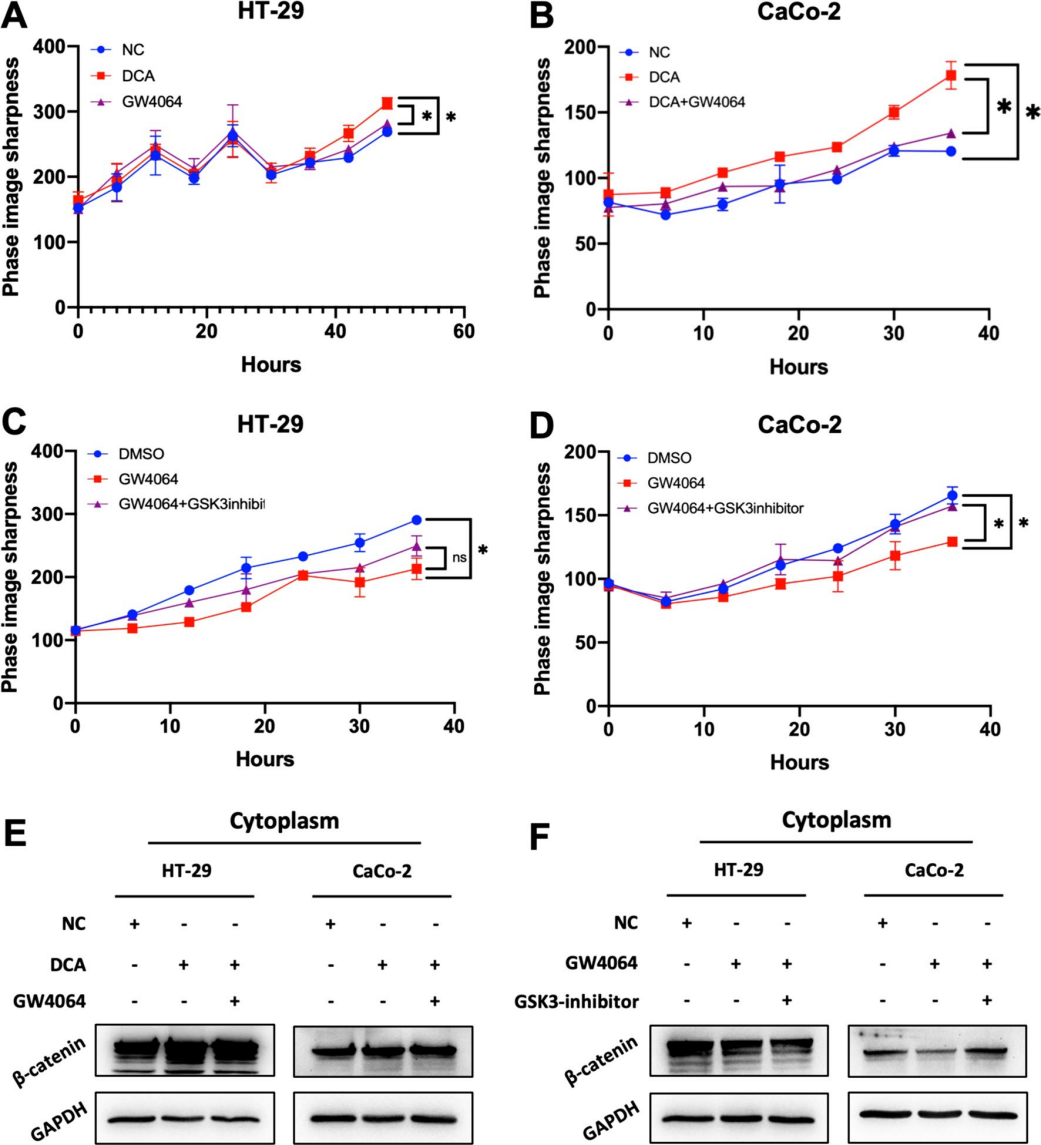
GW4064 and GSK inhibitor rescue the promoting effect and inhibiting effect of DCA and GW4064 by regulating the cytosolic β-catenin expression in CC line cells. **a**, **b** Living-cell image was used to detect the proliferate capability of CC cells treated with DCA and DCA + GW4064. **c**, **d** Living-cell image was used to detect the proliferate capability of CC cells treated with GW4064 and GW4064 + GSK inhibitor. **e** Western blotting was used to detect the protein level of cytosolic β-catenin in CC cells of NC, DCA, DCA + GW4064. **f** Western blotting was used to detect the protein level of cytosolic β-catenin in CC cells of DMSO, GW4064, and GW4064 + GSK inhibitor. *P < 0:05, **P < 0.01, ***P < 0.001

To further test whether a GSK inhibitor rescues GW4064 inhibition of Wnt-related gene and cell proliferation, we performed a live-cell imaging assay and WB to validate it. The results demonstrated that GW4064 suppressed the proliferating capacity of CC line cells, and this inhibitory effect could be rescued by GSK inhibitor. Western blotting showed cytosolic β-catenin was gently increased in GSK-inhibitor rescue groups compared to GW4064 groups (Fig. 6c, d, f). These results indicated that FXR could inhibit the Wnt-β-catenin signaling pathway. Collectively, we proposed that XGB induced an increase in DCA levels and subsequently promotes cell proliferation and cell cycle progression by inhibiting FXR expression and activating the Wnt signaling pathway (Fig. 6).

## Discussion

**Fig. 7:**
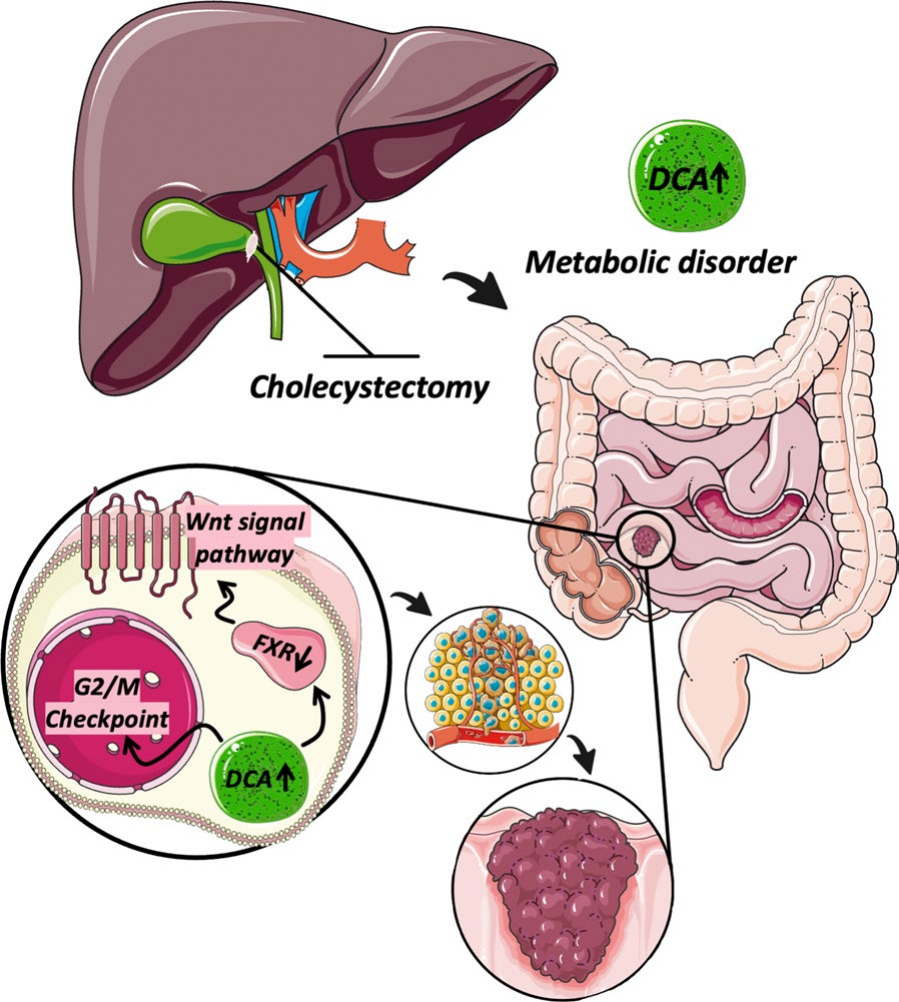
Diagram of molecular mechanism

The safety and efficacy of XGB in the treatment of cholelithiasis have already been well established [24]. Many case–control studies have proposed that XGB increases the risk of CC [9, 25, 26]. Therefore, some colon cancer screening programs even considered XGB as one of the high-risk factors for developing CC development [3, 27, 28]. However, rigorous controlled experiments to establish a causal relationship between XGB and CRC are still lacking. Here we proved for the first time that XGB promotes the formation of CC for the first time in a CAC animal model, which is a fundamental finding for further mechanistic research (Fig. 7).

Previous studies showed that XGB causes the continuous secretion of bile acids, which changes the regularity of bile acid secretion in normal diets and increases the opportunity for bile acid exposure to intestinal flora, leading to an increase in secondary bile acids [29]. DCA is the main component of secondary bile acids. Bayerdorffer et al.[10] demonstrated that plasma DCA levels in patients with colorectal adenoma were significantly increased compared to those in healthy controls. In addition, DCA has been proven to promote the development of a variety of gastrointestinal malignancies [30]. In this pilot study, we performed the XGB surgery on the mice before AOM/DSS-mediated induction of CAC to investigate whether XGB affects the formation and development of CC. The tumor load of the XGB mice was significantly increased compared to that of the sham mice, suggesting a promoting effect of XGB on CAC carcinogenesis. We also observed that the plasma levels of DCA in XGB mice were significantly increased compared to those in sham mice, which suggested that XGB might promote CC tumorigenesis by increasing DCA levels.

It has been reported that changes in DCA levels can disrupt the intestinal mucosa and increase colorectal cancer risk [11, 31]. In this study, we revealed that XGB increased the level of plasma DCA in vivo, and further proved that DCA promoted the proliferation, migration, and the cell cycle of CC cells in vitro*.* Thus, we hypothesized that DCA might accelerate colon carcinogenesis by enhancing the proliferation and migration abilities of colon epithelial cells. To clarify the mechanism by which DCA promotes tumorigenesis, we performed RNA sequencing. The results showed that DCA activated the Wnt signaling pathway and suppressed the expression of G2/M checkpoint genes. Considering that Wnt signaling is the most important pathophysiological pathway for CC, we proposed that the change in the Wnt signaling pathway after DCA treatment could be the main molecular basis of the XGB-induced tumor-promoting effects.

β-Catenin is a crucial protein in Wnt signaling pathway and is mainly located in the cell membrane. APC-Axin-glycogen synthase kinase 3β (GSK-3β) causes β-catenin enters to enter the cytoplasm to be ubiquitinated and degraded. When the Wnt signal is activated and the APC-Axin-GSK3β axis is disrupted, the increases in β-catenin and lymphoid enhancer-binding factor/T-cell factor (LEF/TCF) transcription factors in the nucleus regulate the transcription of downstream target genes (such as c-Myc and cyclinD1) and promote the tumorigenesis [21]. Therefore, we evaluated the Wnt signaling pathway by detecting the expression levels of β-catenin, c-Myc, and cyclin D1 in colon tissues. We found that XGB increased β-catenin, c-Myc, and cyclin D1 expression in CC tissues as compared to noncancerous tissues. We also detected the β-catenin, c-Myc and cyclinD1 expression in CC lines and the results showed that the levels of all of these genes were increased after DCA treatment. In addition, the rescue results indicated that GW4064 significantly inhibited the proliferation of CC line cells treated with DCA, and WB showed cytosolic β-catenin was decreased in GW4064 rescue group compared to DCA group. Therefore, we suggest that Wnt signaling is upregulated by the XGB-induced increase in DCA levels, which could be a possible mechanism by which XGB promotes colonic adenoma-carcinoma formation.

FXR is a nuclear receptor that binds and interacts with DCA in bile acid metabolism [32–34]. Studies have shown that DCA downregulates FXR expression, which is the main driving factor for the development of CC [17]. FXR suppresses CC by antagonizing Wnt/β-catenin signaling [21, 35]. Even though FXR is widely known as a key bile acid receptor, there is no research on whether XGB could cause changes in FXR expression. Here we found that DCA significantly downregulated the expression of FXR and upregulated the expression of Wnt signaling pathway components in CC cell lines. We also confirmed that the FXR agonist GW4064 accelerated cell apoptosis by downregulating the expression of the Wnt signaling pathway. To further validate the effect of GW4064 for Wnt/signaling pathway, we also detected the cytosolic β-catenin in CC line cells treated with DMSO and GW4064, and an inhibitor of GSK3 was used to perform the rescue experiment. The results demonstrated that cytosolic β-catenin was drastically changed, which is in accord with the qPCR assays. Overall, we proposed that XGB could suppress FXR expression by increasing the level of DCA, which subsequently promotes carcinogenesis by activating the Wnt signaling pathway. Considering that GW6064 showed very promising effects in terms of inhibiting cell proliferation and promoting cell apoptosis, we believe that FXR could be a therapeutic target for CC and that GW4064 has great potential for CC prevention or treatment. In addition, to futher validate the Wnt/signaling activation, cytosolic β-catenin was detected in CAC tissues and CC line clles, and it was drastically changed, especially in rescue groups.

We also showed that DCA activates angiogenesis genes and inhibits G2/M checkpoint genes in CC cells. The process of angiogenesis includes the dissolution of the extracellular matrix (ECM) and the proliferation of endothelial cells (ECs), which are largely controlled by a variety of cytokines. Among them, VEGF could enhance the proliferation of ECs and promote the expression of adhesion factors [36–38]. The G2/M checkpoint is the most important cell checkpoint in the cell cycle and represents the last time a cell repairs DNA damage before mitosis. CDK1 is the core element involved in G2/M phase transition and can be inactivated when cell DNA is damaged, eventually leading to cell cycle arrest in the G2 phase [39–41]. In this study, we found that the expression levels of VEGF-A and CDK1 were increased and decreased respectively under DCA treatment. As a nuclear receptor, FXR may regulate the expression of these factors upon DCA treatment, but this hypothesis still needs to be further explored.

In conclusion, XGB promotes the carcinogenesis and progression of CC by increasing plasma DCA levels. Mechanistically, DCA activates the Wnt-β-catenin signaling pathway by decreasing the expression of FXR.

## Data Availability

The datasets and materials used for the study are available from the corresponding author on reasonable request.

## Abbreviations

CAC: Colitis-associated CC
CC: Colon cancer
DCA: Deoxycholic acid
DEGs: Differentially expressed genes
ECM: Extracellular matrix
ECs: Endothlial cells
EdU: 5-Ethynyl-2’-deoxyuridine
FBS: Fetal bovine serum
FITC: Fluorescein isothiocyanate
FXR: Farnesoid X receptor
IHC: Immunohistochemistry
LEF/TCF: Lymphoid enhancer-binding factor/T cell factor
MS: Mass spectrometry
PI: Propidium iodide
PPI: Protein–protein interaction
qRT-PCR: Quantitative real-time
UPLC: Ultra performance liquid chromatography
XGB: Cholecystectomy
