# Correction: Cholecystectomy promotes colon carcinogenesis by activating the Wnt signaling pathway by increasing the deoxycholic acid level

**DOI:** 10.1186/s12964-022-00908-1

**Published:** 2022-06-13

**Authors:** Yuxia Yao, Xiangji Li, Baohong Xu, Li Luo, Qingdong Guo, Xingyu Wang, Lan Sun, Zheng Zhang, Peng Li

**Affiliations:** 1 Department of Gastroenterology, Beijing Friendship Hospital, Capital Medical University, National Clinical Research Center for Digestive Disease, Beijing Digestive Disease Center, Beijing Key Laboratory for Precancerous Lesion of Digestive Disease, Beijing, 100050 People’s Republic of China; 2 Department of Gastroenterology, Beijing Luhe Hospital, Capital Medical University, Beijing, 101149 People’s Republic of China; 3 Innovation Laboratory of Terahertz Biophysics, National Innovation Institute of Defense Technology, Beijing, 100071 People’s Republic of China; 4 Department of Retroperitoneal Tumor Surgery, Peking University International Hospital, Beijing, 102206 People’s Republic of China; 5 Department of Pathology, Beijing Luhe Hospital, Capital Medical University, Beijing, 101149 People’s Republic of China

## Correction to: Cell Communication and Signaling (2022) 20:71 10.1186/s12964-022-00890-8

Following publication of the original article [1], the authors identified an error in Fig. 6E. The updated Fig. 6 is given in this correction article. The original article [1] has been corrected.

**Fig. 6:**
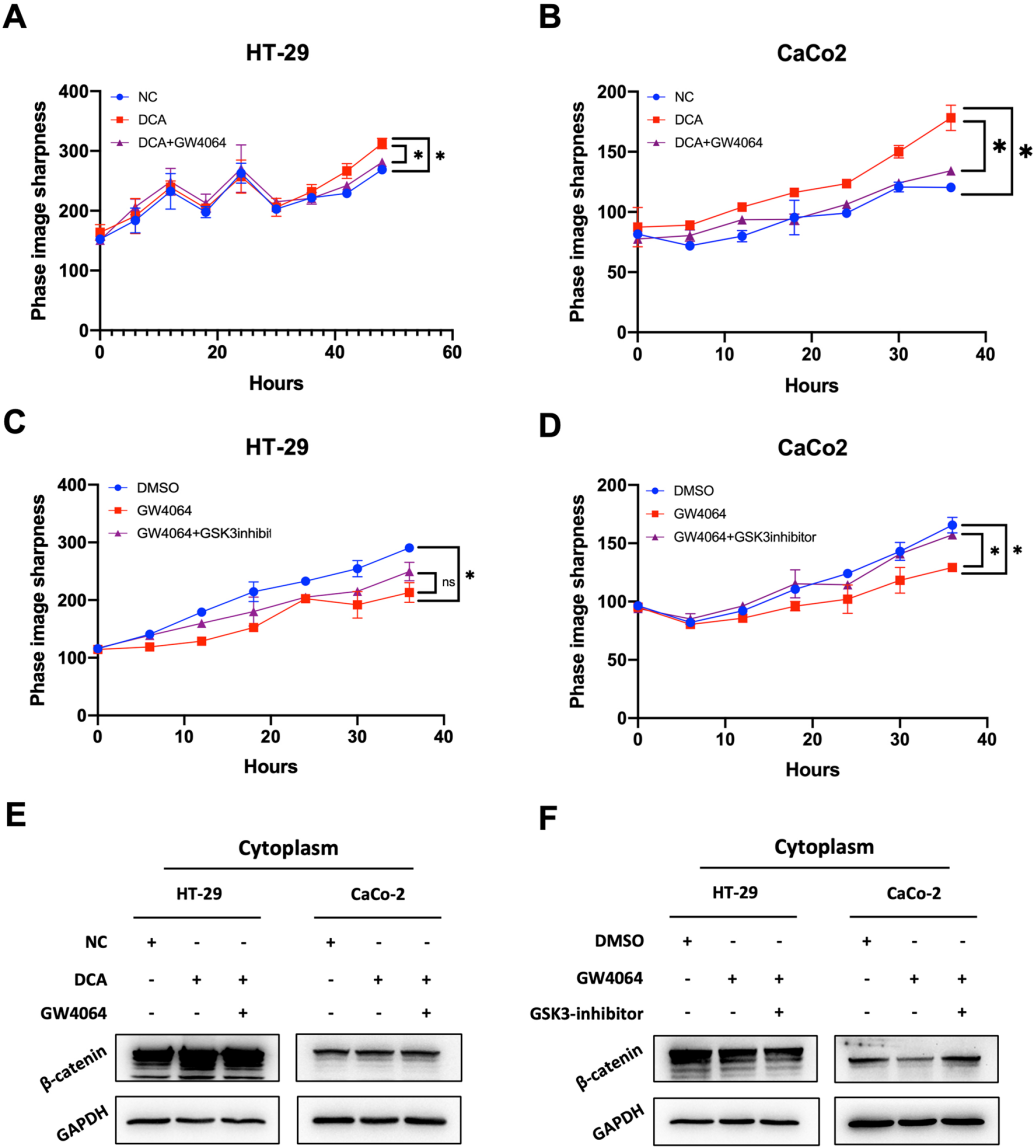
GW4064 and GSK inhibitor rescue the promoting effect and inhibiting effect of DCA and GW4064 by regulating the cytosolic β-catenin expression in CC line cells. **a**, **b** Living-cell image was used to detect the proliferate capability of CC cells treated with DCA and DCA + GW4064. **c**, **d** Living-cell image was used to detect the proliferate capability of CC cells treated with GW4064 and GW4064 + GSK inhibitor. **e** Western blotting was used to detect the protein level of cytosolic β-catenin in CC cells of NC, DCA, DCA + GW4064. **f** Western blotting was used to detect the protein level of cytosolic β-catenin in CC cells of DMSO, GW4064, and GW4064 + GSK inhibitor. *P < 0:05, **P < 0.01, ***P < 0.001
